# Both Isolated Long Head of the Biceps Tenotomy and Tenodesis Are Effective for Symptomatic Rotator Cuff Repair Revision

**DOI:** 10.3390/jcm14030852

**Published:** 2025-01-28

**Authors:** Alessander D’Ascoli, Edoardo Giovannetti de Sanctis, Nicolas Bronsard, Marc-Olivier Gauci, Jean-François Gonzalez

**Affiliations:** 1 IULS—Institut Universitaire Locomoteur et Sports, Pasteur 2 Hospital, CHU, 06000 Nice, France; alessander.dascoli@gmail.com (A.D.);; 2 Unité de Recherche Clinique (UR2CA), Université de Nice Côté d’Azur, 06000 Nice, France

**Keywords:** biceps tenodesis, biceps tenotomy, long head biceps, revision, rotator cuff repair

## Abstract

**Background:** Symptomatic rotator cuff (RC) repair continues to be a complex issue. Leaving the long head of the biceps (LHB) in place might increase the risk of residual pain, even in the case of a healed RC. The purpose of this study was to assess the clinical outcomes of isolated LHB tenotomy and tenodesis as a revision procedure in symptomatic patients that had previously undergone an arthroscopic RC repair with no clinical or MRI evidence of RC retear. **Methods:** A retrospective analysis was conducted on patients with a persisting painful shoulder after an arthroscopic RC repair with no clinical or MRI signs of cuff retear, undergoing an isolated arthroscopic biceps tenotomy or tenodesis as a revision procedure. Functional outcomes were assessed preoperatively and at a minimum of 24 months of follow-up. **Results:** A total of 88 patients were included. The biceps tendon was managed with biceps tenodesis in 64 patients and tenotomy in 24 patients. VAS, Constant Score, SSV and active anterior elevation were all significantly improved after revision surgery. There was no significant difference between pre- and postoperative anterior passive elevation. No significant difference was shown between the tenodesis and tenotomy groups. **Conclusions:** The present study demonstrated that both isolated tenotomy and tenodesis are effective and safe in treating patients with a symptomatic shoulder after RC repair at a 2-year follow-up with a very low complication rate. Although tenodesis did not show any significant clinical benefit outcomes compared to tenotomy, it might be associated with a lower risk of Popeye deformity.

## 1. Introduction

Although rotator cuff repair (RCR) is an effective procedure with encouraging clinical results, some patients may show persistent pain and impaired shoulder function [1].

Symptomatic rotator cuff repair continues to be a complex issue for treating surgeons. The most frequent cause of failure is rotator cuff retear, whose occurrence has been shown to vary between 10 and 94% [1].

A potential alternative cause of failure and therefore an important variable during RCR is the management of the long head of the biceps (LHB) tendon [2].

The LHB has been shown to be a common shoulder pain generator, causing severe disability [3]. Patients with rotator cuff tears have a high incidence of concomitant long head of the biceps tendon pathology (with a range from 16 to 75%), which may then be responsible for shoulder pain and dysfunction [4,5,6]. This is due to the closeness of the LHB and its sheath to the RC, which makes it susceptible to any inflammatory process affecting the latter [6].

Biceps pathology may then occur due to the failure of concomitant procedures, such as a rotator cuff retear, and be the cause of a symptomatic rotator cuff repair requiring revision [3].

Leaving the LHB in place might increase the risk of residual pain, even in the case of a healed RC. The rate of LHB procedures associated with RCR has increased each year, showing improvement in the awareness of biceps function and pathology [7].

Arthroscopic LHB tenotomy and tenodesis have become popular in the last two decades as treatment options for patients with painful degenerative rotator cuff tears with early pain relief [4,6,8,9,10,11,12].

However, there continues to be an ongoing debate on whether or not to systematically treat the LHB during an RCR [2,5,7,13,14,15], and there is still no consensus on which technique is superior between tenotomy and tenodesis [16,17,18,19,20]. Furthermore, the question of which technique should be used while performing LHB tenodesis is controversial, in terms of the type of fixation (interference screw or anchors) and the location of tenodesis (intra-articular or extra-articular; superior or inferior to the pectoralis major insertion) [21,22,23,24,25,26,27,28,29,30].

To our knowledge, there are no data on the outcomes of an isolated LHB surgical treatment for persistent pain after a prior healed rotator cuff repair.

The purpose of this study was to assess the clinical outcomes of isolated LHB tenotomy and tenodesis as a revision procedure in symptomatic patients that had previously undergone an arthroscopic rotator cuff repair with no clinical or MRI evidence of RC retear.

Our main hypothesis was that both techniques significantly improve clinical outcomes. Our second hypothesis was that arthroscopic tenodesis would have fewer complications than but a similar satisfaction rate to arthroscopic tenotomy.

## 2. Patients and Methods

### 2.1. Study Overview and Design

After receiving institutional review board approval for this study (Ref.1-2024), a retrospective analysis of a prospectively maintained institutional database was conducted at the Hopital Pasteur II-CHU de Nice (France), searching for patients with a painful shoulder after an arthroscopic rotator cuff repair, being revised between 2006 and 2020.

Patients were included in this study if they had undergone an isolated arthroscopic biceps tenotomy or tenodesis as a revision of a previously performed rotator cuff repair.

The exclusion criteria were a preoperative (MRI) or intraoperative diagnosis of RC retear.

**Figure 1:**
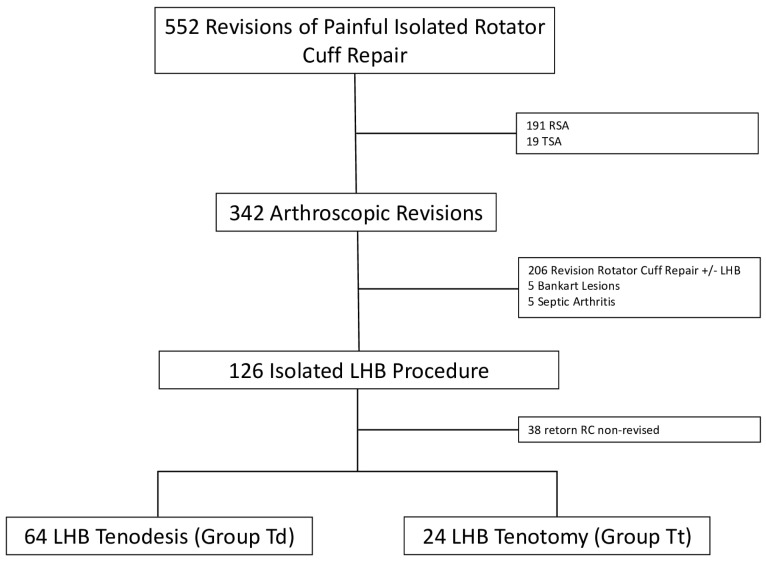
Flow-chart detailing patient selection process.

A total of 552 patients presented with a painful shoulder after an arthroscopic cuff repair (Figure 1). A total of 210 patients showed signs of gleno-humeral osteoarthritis or massive irreparable RC and had a shoulder replacement. A total of 206 patients presented with a reparable RC retear and underwent an arthroscopic rotator cuff repair revision. Ten patients were further excluded, having either an associated Bankart lesion or signs of shoulder arthritis.

A total of 126 patients underwent an isolated LHB procedure. However, intraoperatively, 38 patients showed evidence of a small retorn RC which was not repaired and were therefore excluded. A total of 88 patients were then included in this study, undergoing an isolated tenotomy or tenodesis of the LHB associated with a subacromial bursectomy.

All the patients had undergone the first arthroscopic surgery outside of the CHU-Nice as the systematic tenotomy or tenodesis of LHB associated with a rotator cuff repair was performed in our center.

The indications for an isolated LHB arthroscopic procedure were the persistence of shoulder pain and either positive LHB tests (e.g., Palm-up and O’Brien tests) or MRI findings but negative rotator cuff weakness assessed with the Jobe Test with no MRI signs of cuff retear after a minimum of one year following primary rotator cuff repair.

### 2.2. Radiographic and Clinical Evaluation

Each patient was evaluated preoperatively with both a radiograph, to assess the presence of osteoarthritis and Cuff Tear Arthropathy (CTA) according to the Hamada Classification [31], and MRI (Magnetic Resonance Imaging) to exclude rotator cuff retear.

All patients showed MRI signs of LHB tenosynovitis, defined as an abnormal-appearing (heterogeneous appearance) tendon and a diffused distending sheath effusion. No patients included within this cohort had clear biceps subluxation or slap tear. All MRIs were reviewed by the first author.

During surgery, the RC was systematically checked to assess the presence of a retorn RC. The position of the biceps in the bicipital groove and global aspect were reported. For the 88 included patients, the preoperative imaging diagnosis of LHB tenosynovitis was confirmed by the intraoperative findings. LHB tenodesis was usually proposed for physically active men and tenotomy for elderly patients or women with low physical activity levels.

Functional outcomes were assessed with ROM (passive and active anterior elevation), the Constant–Murley Score (CS) [32], the subjective shoulder value (SSV) score [33] and the Visual Analog Scale (VAS) score for pain preoperatively and at a minimum of 24 months of follow-up. The last follow-up was performed by the first author for all patients.

### 2.3. LHB Tenodesis Technique

The procedure is performed with a 30° arthroscope with the patient in the beach chair position with the elbow kept in extension. While viewing from the posterior portal, a spinal needle is inserted through the intra-articular portion of the LHB tendon. A radiofrequency ablation device or an arthroscopic cutting instrument is then introduced through the anterior portal to perform the LHBT tenotomy.

The arthroscope is then placed through the lateral portal within the subacromial space. An electrocautery ablation device is then introduced through the anterolateral portal, and the subacromial bursa is removed to obtain a panoramic view of the subdeltoid space. The same device is then used to completely release the transverse ligament along the bicipital groove. An anchor (Y-Knot RC all-suture anchor; ConMed, Largo, FL, USA) is placed within the upper portion of the bicipital groove. Both sutures loaded within the anchor are used for the tenodesis technique, preventing the tendon from retracting into the bicipital groove.

The first suture is grasped by using the jaw of the Cleverhook grasper and is pulled back through the LHBT, but not completely through, leaving a loop. The tip of the Cleverhook is then passed through the loop to grasp the free end of the suture which is pulled through the loop.

The second suture is grasped and pulled medially to the LHBT, leaving a loop. The tip of the Cleverhook is similarly passed through the loop to grasp the free end of the suture. Each of the sutures are then tightened separately.

The portion of the LHBT superior to the suture knots is then cut with an electrocautery device, and the intra-articular remnant of the LHBT is then removed with a grasper.

### 2.4. Statistical Analysis

IBM SPSS ver 29.0 Software (IBM Corp., Armonk, NY, USA) was used to carry out statistical analysis. Linear regression models were used to assess comparability between groups. VAS, CS, SSV and ROM in shoulders in passive and active elevation before and after the LHB procedure were compared with Student’s *t*-test with a significance level of 0.05. A Wilcoxon rank sum test was used to compare the postoperative results between the Td and Tt groups with a significance level of 0.05. The statistical analysis was performed by the first author.

## 3. Results

The biceps tendon was managed with biceps tenodesis in 64 patients (Td group) and tenotomy in 24 patients (Tt group).The mean age and mean follow-up were, respectively, 56 ± 8.24 years and 62 months. There were 56 (64%) men and 32 (36%) women. The mean time interval between RCR and revision was 6.2 years (Table 1).

There was no significant difference in any of the demographic and clinical characteristics of the patients between the Td and Tt groups, except for age and sex. Patients in Td group were found to be younger and predominantly males compared to Tt.

The LHB procedure significantly improved postoperative shoulder pain and function in terms of VAS, Constant Score, SSV and active anterior elevation (*p* < 0.05) (Table 2).

These results were similar in the Td and Tt subgroups. No significant difference was found between Td and Tt concerning preoperative shoulder function and pain. Age and sex might be confounding factors for these results. Patient satisfaction, which was the subject of our second hypothesis assessed by the SSV score, showed no significant difference in both subgroups (Table 3).

The study population was classified in different stages according to Hamada et al. [31]. There were no significant differences between the groups when examining patient radiographs according to the Hamada Classification (*p* = 0.725). No patients showed clinical or radiographic signs of significant osteoarthritis.

There was no significant difference in anterior passive elevation (*p* > 0.05) either in the tenodesis or tenotomy subgroup (Table 2).

There was a total of two (1.5%) cases of postoperative capsulitis, requiring arthrographic distension (one within each group). Six patients underwent a Reverse Shoulder Arthroplasty (RSA) (two patients in Td group and four patients in Tt group) after 4.3 years.

Six patients who underwent a biceps tenodesis and six patients who underwent a biceps tenotomy had a postoperative Popeye sign (*p* = 0.081).

## 4. Discussion

The most important finding of this study was that LHB tenodesis or tenotomy as an isolated revision surgery is an effective, reproducible and safe procedure for a painful impaired shoulder in a population with a history of arthroscopic RCR without any associated LHB procedures and with no RCR retear, significantly improving pain and functional scores at a 2-year follow-up.

The impact that the LHB pathology has on the symptoms of patients with RC tear is still debated.

The data from this study suggest that a concomitant LHB procedure performed systematically while treating patients for RC tears might decrease the risk of persistent pain and suboptimal outcomes after the repair. However, since the entire study population had undergone the first procedure in other institutions, it is difficult to evaluate the number of patients presenting with persisting pain after arthroscopic RCR without a concomitant LHB procedure.

To date, a few authors have examined RCR with and without a biceps procedure, showing controversial results [2,15,34].

Watson et al. assessed the outcomes of 80 patients at a 1-year follow-up, comparing an isolated RCR to an LHB procedure concomitant with the RCR [6]. The use of a concomitant LHB procedure demonstrated a greater improvement in patient-reported outcome measures and a better ASES score at the 1-year follow-up.

Nemirov et al. evaluated the role of a concomitant biceps intervention in rotator cuff repair [34]. The authors showed similar outcomes and complication/revision rates when comparing isolated RCR and RCR with a biceps procedure, either tenodesis or tenotomy.

Baumgarten et al. compared patients undergoing RCR with and without biceps tenodesis [15]. The indication for a biceps procedure was a partial LHB lesion or biceps instability. No significant difference in postoperative clinical outcomes was shown.

Erickson et al. stated that concomitant biceps tenodesis might increase the risk of reoperation after rotator cuff repair [2]. The authors therefore suggested preserving the biceps when its contribution to shoulder pain is uncertain. However, reoperation was defined, differently from our study, as a second rotator cuff surgery.

This is the first study assessing the results of an isolated LHB procedure as a revision surgery, in treating patients with a history of RC repair. However, the literature evaluating the clinical outcomes of the same procedure as a primary surgery for RC pathology is quite limited.

Walch et al. evaluated 307 LHB tenotomies not associated with RCR at a mean of 57 months of follow-up, highlighting a high satisfaction rate (87%) [10].

Boileau et al. retrospectively assessed 68 consecutive patients with an irreparable RC lesion treated arthroscopically with biceps tenodesis or tenotomy at a mean follow-up of thirty-five months [4]. Both treatments were effective in treating severe pain and dysfunction, with no statistically significant difference between the procedures.

Veen et al. evaluated the outcomes of 64 patients undergoing an arthroscopic primary isolated biceps tenotomy for degenerative rotator cuff tear, showing a perceived improvement in pain at 4.2 years of follow-up in 75% of patients [35].

Descamps et al. in a systematic review demonstrated significant improvements in Constant–Murley and pain scores after isolated biceps tenotomy or tenodesis for massive irreparable tears.

Our data suggest that patients with a symptomatic rotator cuff repair can be informed that an isolated LHB procedure might significantly improve their clinical outcomes at 2 years.

Our study’s complication rate is quite low, even though two patients complicated with capsulitis, leading to a stiff shoulder, requiring a radiologic dilatation [8].

No statistically significant differences in clinical outcome scores were shown between tenodesis and tenotomy, even though age could be a confounding factor. The subjective shoulder value was not significatively altered, confirming our secondary hypothesis.

The present study does not prove any clinical superiority of one of the techniques. Elbow flexion and supination strength were not evaluated, limiting the data to be compared.

It is still debated whether tenodesis might lead to better clinical outcomes in terms of function and pain [36,37,38,39].

Tenotomy is an easier and faster technical procedure associated with the earlier relief of pain and return to activity and fewer restrictions after the procedure but with a greater risk of postoperative Popeye deformity and arm cramping [16,17,38]. Conversely, tenodesis is associated with a longer surgical time and a higher cost of implants but with a lower risk of Popeye deformity and potentially greater supination strength [16,17,39]. Surprisingly, there was no significant difference for Popeye deformity between the Td and Tt groups.

Both techniques are acceptable and successful options while treating patients with painful rotator cuff repair without a history of LHB procedures. The decision should be based on patient characteristics and expectations (e.g., physical activity, cosmesis, recovery period) to individualize treatment and potentially increase patient satisfaction [36].

This study has several limitations. This is a retrospective, nonrandomized review of patients. A slight difference in sample characteristics and size exists between the two groups (Td and Tt). The physical exam specific to the LHB does not include any elbow flexion and supination strength data.

Ultrasound or other diagnostic tools were not used postoperatively to assess the status of the biceps procedure. Tenodesis integrity might be doubtful in some patients as it was not evaluated with postoperative imaging, and six patients within the tenodesis group showed a Popeye deformity. It is therefore reasonable to think that some of the patients considered within the tenodesis group altered the results, having had a spontaneous postoperative LHB tendon rupture.

We believe that our study has many potential strengths. To the best of our knowledge, the present study is the first to report the outcomes of isolated LHB tenotomy/tenodesis for treating a symptomatic rotator cuff repair. In addition, the sample size for this specific isolated procedure as a revision surgery is relatively large, and all patients who underwent this procedure in our center were included.

## 5. Conclusions

This study demonstrated that both isolated tenotomy and tenodesis are effective and safe in treating patients with a symptomatic shoulder after rotator cuff repair without an initial procedure conducted on the LHB tendon, being assessed at a 2-year follow-up with a very low complication rate.

Although tenodesis did not show any significant clinical benefit outcomes compared to tenotomy, it might be associated with a lower risk of Popeye deformity.

## Figures and Tables

**Table 1:** Patient characteristics. Td: tenodesis; Tt: tenotomy; yrs: years; M: male; F: female.

	Total	Td Group	Tt Group	*p* Value
Patients	88	64	24	
Age (yrs)	56 ± 8.24	53.4 ± 7.46	61.8 ± 7.23	<0.001
Sex (M/F)	50/38	42/22	16/8	0.006
Dominant (%)	53 (47/88)	55 (35/64)	50 (12/24)	0.695
Professional activity (%)	80 (70/88)	77 (49/64)	88 (21/24)	0.376
Smoker (%)	16 (14/88)	16 (10/64)	17 (4/24)	1
Diabetes (%)	3.4 (3/88)	4.7 (3/64)	0 (0/24)	0.559
Time to revision (yrs)	6.2 ± 3.02	6.3 ± 3.00	5.7 ± 3.06	0.358

**Table 2:** Comparison of preoperative and postoperative outcome scores in entire cohort. VAS: Visual Analog Score; SSV: Simple Shoulder Value; AAE: Anterior Active Elevation; APE: anterior passive elevation.

	Preoperative	Postoperative	Mean Difference	*p* Value
VAS	7.03 ± 1.30	2.61 ± 1.93	−4.42	<0.001
Constant Score	52.5 ± 10.5	68.5 ± 12.2	+16	<0.001
SSV	45 ± 17.8	64.8 ± 14.2	+19.8	<0.001
AAE	134 ± 24.0	148 ± 18.4	+14	<0.001
APE	160 ± 15.5	160 ± 15.1	null	0.103

**Table 3:** Comparison of preoperative and postoperative outcomes between tenodesis and tenotomy. VAS: Visual Analog Score; CS: Constant Score; SSV: Simple Shoulder Value; AAE: Anterior Active Elevation; APE: Anterior Passive Elevation; M.I.: Mean Improvement.

	Preoperative			Postoperative				
	Td Group	Tt Group	*p* Value	Td Group	M.I.	Tt Group	M.I.	*p* Value
VAS	7.00 ± 1.30	7.12 ± 1.33	0.695	2.56 ± 1.74	−4.44	2.75 ± 2.42	−4.37	0.860
CS	52.8 ± 10.8	51.7 ± 9.78	0.646	68.6 ± 12.6	+15.8	68.1 ± 11.1	+16.4	0.853
SSV	44.8 ± 17.9	45.6 ± 17.8	0.842	64.6 ± 14.9	+19.8	65.4 ± 12.6	+19.8	0.875
AAE	135 ± 22.2	131 ± 28.6	0.516	149 ± 17.3	+14	146 ± 21.2	+15	0.792
APE	161 ± 14.0	156 ± 18.6	0.242	161 ± 14.0	null	157 ± 17.8	+1	0.305

## Data Availability

Data can be obtained by mailing Alessander D’Ascoli.
